# Supplementary material for: PI3K GOF leads to dysregulation of T and B cells that both contribute to extrinsically driving activation and differentiation of other CD4 + T cells
Source: Immunol Cell Biol. 2025 Sep 14;103(9):884–96. doi: 10.1111/imcb.70058 (PMC12521957; doi:10.1111/imcb.70058)
Supplement: Supplementary file 1 — Supplementary data 1 [file IMCB-103-884-s001.pdf]

Supporting information

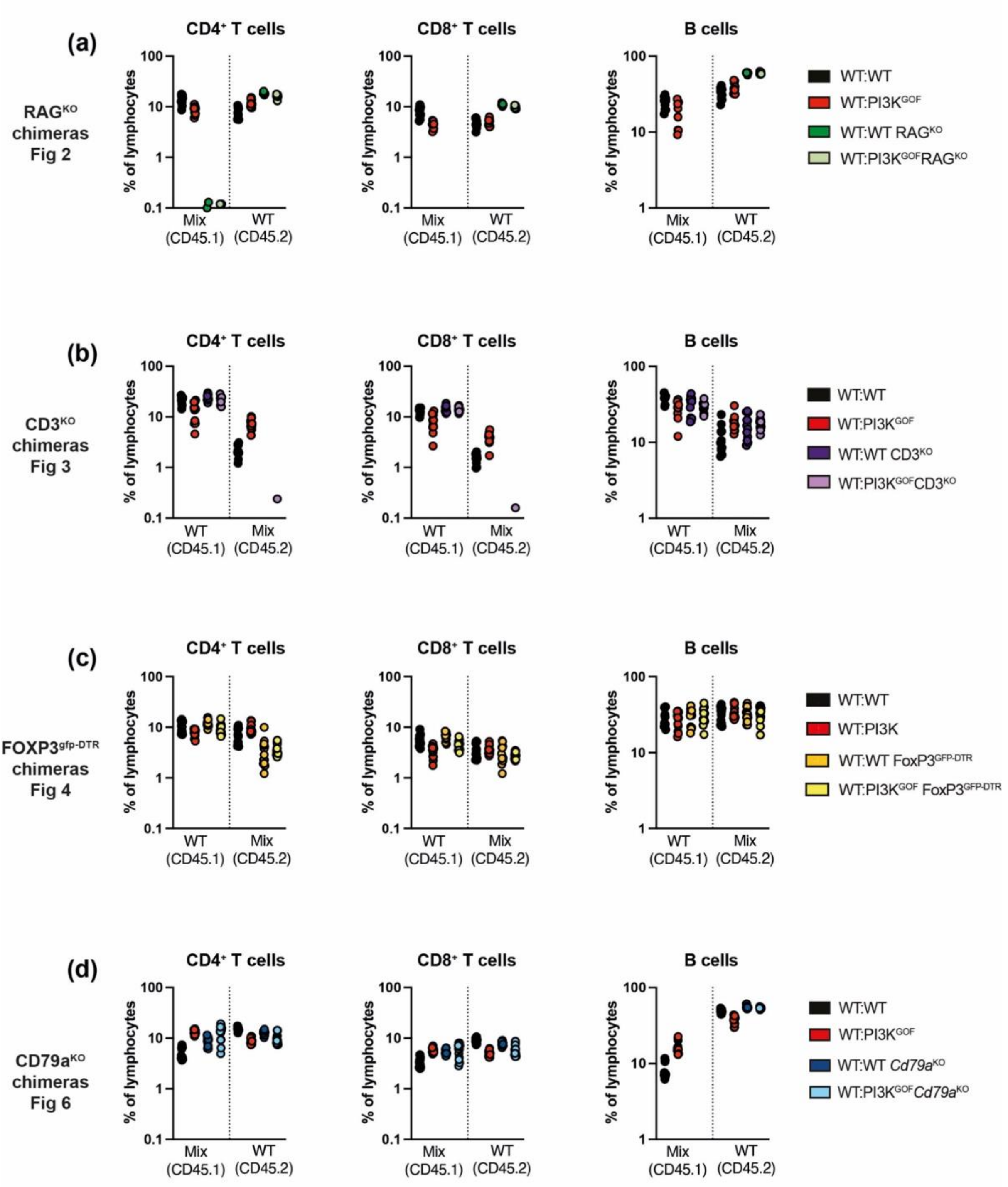

Supplementary figure 1. Reconstitution of T and B cells in chimeras

Mixed chimeras were set up as in (a) Figure 2, (b) 3, (c) 4 and (d) 6. Spleens from mixed chimeras were stained to identify $CD4^+$ and $CD8^+$ T cells and $B220^+$ B cells within the $CD45.1^+$ or $CD45.2^+$ compartments. Each point represents $CD45.1^+$ or $CD45.2^+$ cells in a different mouse.

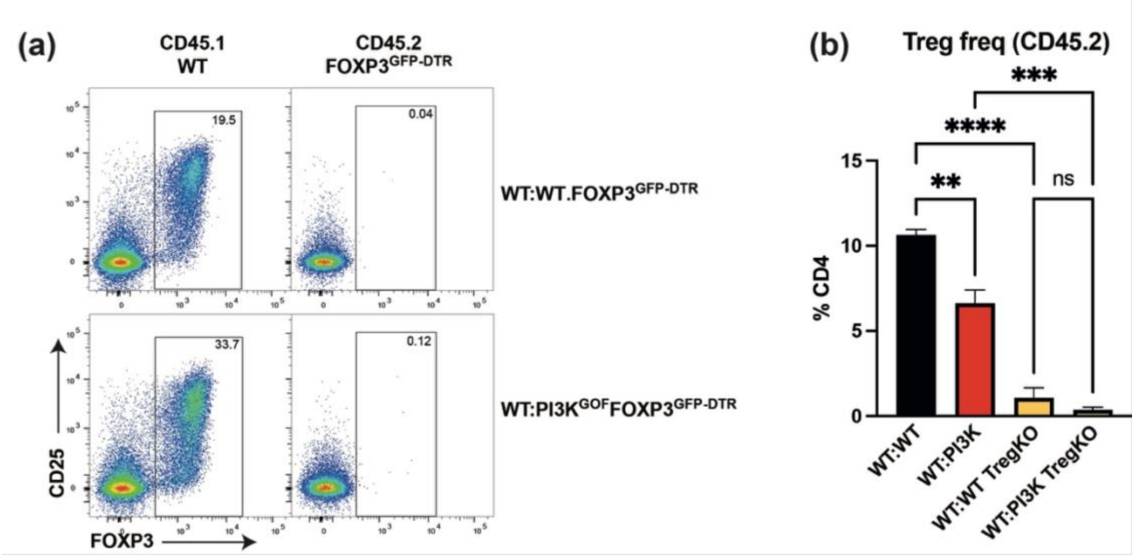

Supplementary figure 2. Depletion of FoxP3$^{GFP-DTR}$ Tregs

Mixed chimeras were set up as in Figure 4A and treated with diphtheria toxin as shown in Fig 4B. Spleens from mixed chimeras were stained to identify CD4$^+$FoxP3$^+$ T cells within the CD45.1$^+$ or CD45.2$^+$ compartments. **(a)** Representative flow plots are shown. **(b)** Plot shows the number of FoxP3$^+$ cells within CD4 T cells in the CD45.2$^+$ compartment in each bone marrow chimera combination. Treg (FoxP3$^+$) CD4$^+$ T cells were determined (bars show means ± SEM, n = 12-13 - data pooled from 2 independent lots of chimeras). Significant differences were determined by using Brown-Forsythe and Welch ANOVA tests: $^{**}P < 0.01$, $^{***}P < 0.001$, and $^{****}P < 0.0001$, ns – not significant.

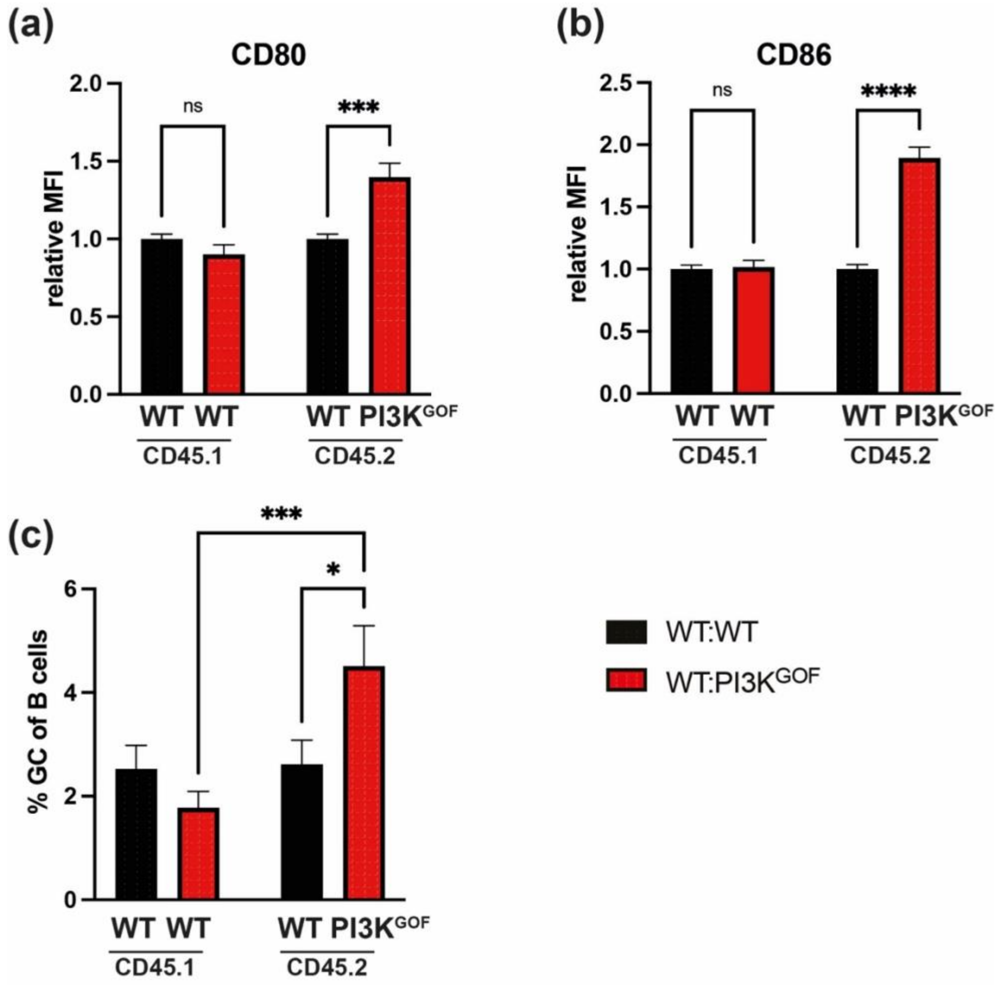

Supplementary figure 3. PI3K GOF drives cell intrinsic activation of B cells

WT:WT or WT:PI3KGOF mixed BM chimeras were generated as shown in Fig 1A. Spleens from 11-15 weeks after reconstitution were stained to identify B220$^+$ B cells within the CD45.1$^+$ or CD45.2$^+$ compartments. The expression of (**a**) CD80 and (**b**) CD86 was determined. Plots show MFI relative to WT cells in chimeras (mean ± SEM, n = 5). **(c)** The percentage of germinal centre (GC) B cells was determined (mean ± SEM, n = 26-27). Significant differences were determined by 2-way ANOVA: *P < 0.05, ***P < 0.001, and ****P < 0.0001, ns – not significant.

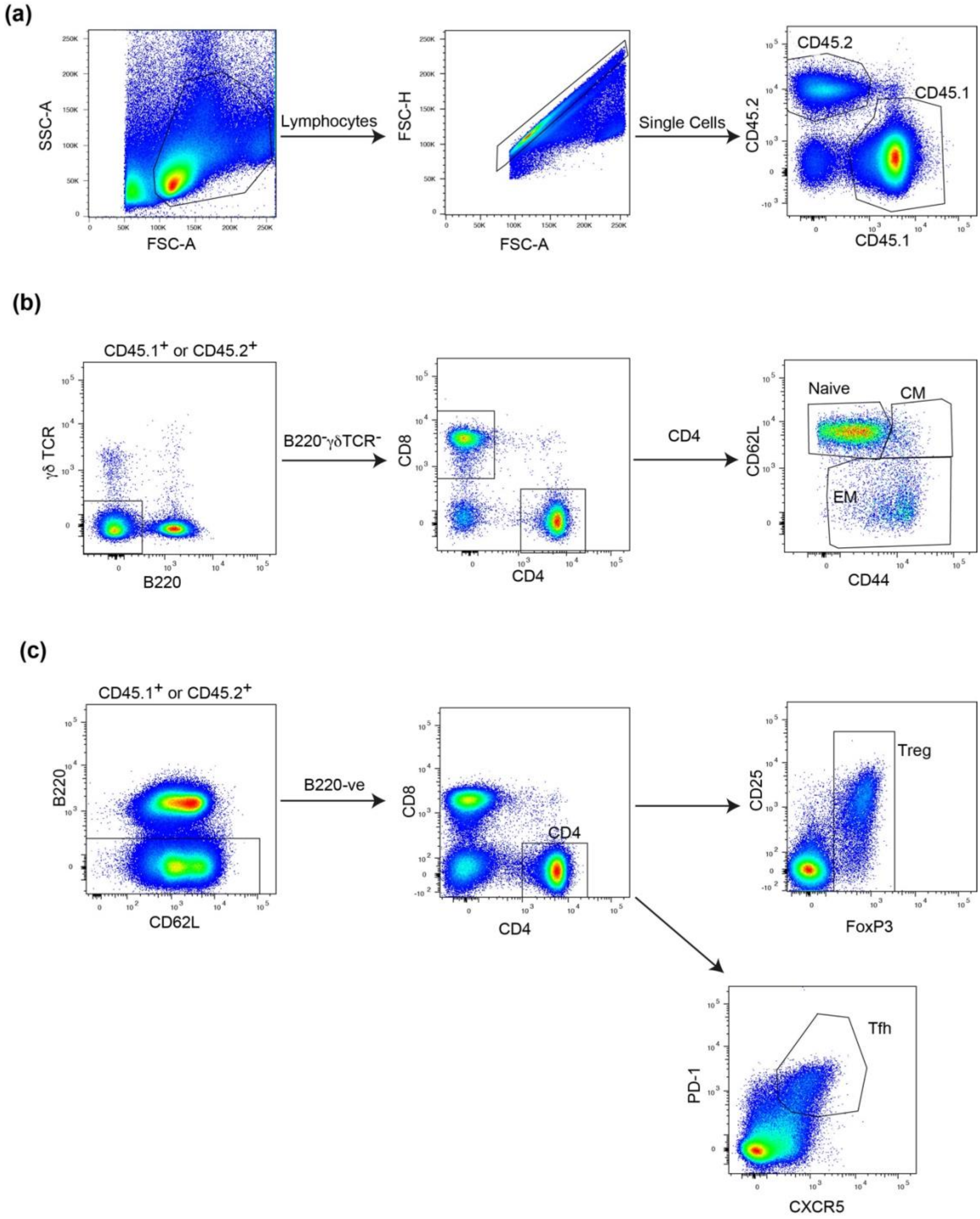

Supplementary figure 4. Gating strategy for identification of CD4 populations. Representative plots for gating strategy (**a**) Lymphocytes were gated, followed by gating on single cells, and then on CD45.1^{+} or CD45.2^{+} (**b**) Cells were gated as B220-γδTCR- to exclude B cells and γδ T cells cells, then on CD4+ and then on naïve, central memory (CM) and effector memory (EM) based on CD62L and CD44. **(c)** To identify Tfh and Treg cells, cells were gated as B220^{-} to exclude B cells and then gated on FoxP3 and CD25 (Tregs) or CXCR5 and PD-1 (Tfh cells).

Supplementary table 1. Gene sets upregulated in Treg #2 population

Gene sets enriched in Treg #2	NES	NOM p-val	FDR q-val	FWER p-val
LI_INDUCED_T_TO_NATURAL_KILLER_UP	2.44	0	0	0
GOLDRATH_ANTIGEN_RESPONSE	2.43	0	0	0
GAVIN_FOXP3_TARGETS_CLUSTER_P3	2.32	0	0	0
GAVIN_FOXP3_TARGETS_CLUSTER_P6	2.32	0	0	0
HESS_TARGETS_OF_HOXA9_AND_MEIS1_DN	2.25	0	0	0
KIM_GLIS2_TARGETS_UP	2.22	0	0	0
MARSON_FOXP3_CORE_DIRECT_TARGETS	2.17	0	0	0
GAVIN_FOXP3_TARGETS_CLUSTER_P4	2.14	0	0	0
MARSON_FOXP3_TARGETS_UP	2.14	0	0	0
KHETCHOUMIAN_TRIM24_TARGETS_UP	2.14	0	0	0
BOYLAN_MULTIPLE_MYELOMA_C_D_DN	2.12	0	0	0.001
GAVIN_FOXP3_TARGETS_CLUSTER_T4	2.11	0	0	0.001
PLASARI_TGFB1_TARGETS_10HR_UP	2.1	0	0	0.001
SAFFORD_T_LYMPHOCYTE_ANERGY	2.07	0	0	0.002
GESERICK_TERT_TARGETS_DN	2.07	0	0	0.002
REACTOME_CLASS_A_1_RHODOPSIN_LIKE_RECEPTORS	2.05	0	0	0.004
VILIMAS_NOTCH1_TARGETS_UP	2.04	0	0	0.005
PLASARI_TGFB1_TARGETS_1HR_UP	2.01	0	0.001	0.011
SEKI_INFLAMMATORY_RESPONSE_LPS_UP	1.99	0	0.001	0.018
BROWN_MYELOID_CELL_DEVELOPMENT_UP	1.99	0	0.001	0.02
GALINDO_IMMUNE_RESPONSE_TO_ENTEROTOXIN	1.98	0	0.001	0.025
FOSTER_KDM1A_TARGETS_UP	1.97	0	0.001	0.028
BIOCARTA_CASPASE_PATHWAY	1.97	0	0.001	0.033
WP_TYROBP_CAUSAL_NETWORK_IN_MICROGLIA	1.97	0	0.001	0.034
QI_PLASMACYTOMA_UP	1.96	0	0.001	0.036
REACTOME_GPCR_LIGAND_BINDING	1.96	0	0.001	0.036
BORLAK_LIVER_CANCER_EGF_UP	1.95	0	0.002	0.05
